# Supplementary material for: On reconstructing Giraffa sivalensis, an extinct giraffid from the Siwalik Hills, India
Source: PeerJ. 2015 Aug 4;3:e1135. doi: 10.7717/peerj.1135 (PMC4540016; doi:10.7717/peerj.1135)
Supplement: Table S1 [file peerj-03-1135-s002.docx]

**Supplementary Table S1 Fossil discoveries which have been assigned to *Giraffa sivalensis***

| **Specimens ascribed to *G. sivalensis*** | **Vertebral fossil specimen: vertebrae** | **Museum no** | **Origin** | **References to specimen** | **Paper summary or highlights** |
| --- | --- | --- | --- | --- | --- |
|  | Cervical vertebra, complete, well preserved, holotype. 'Third' (Falconer & Cautley, 1843) or 'fifth' (Lydekker, 1883) cervical. |  | Siwalik hills, India | Cautley, 1838 | Dimensions of vertebra given - postulated to be Giraffid in origin. |
|  |  |  |  | Falconer & Cautley, 1843 | Detailed description of vertebra and assigned the name *Camelopardalis sivalensis*. Postulated that these animals were in length about a third shorter than modern giraffes. |
|  |  |  |  | Murchison, 1868a (Unpublished plate E, Fig. 1) | Third cervical vertebra of fossil giraffe, from the Siwalik hills. The elongated character of the vertebra shows that the animal had a columnar neck, and the fact that the transverse processes are provided with foramina for the vertebral arteries shows that it was not a camel. The complete synostosis of the upper and lower articulating surfaces, the strong relief of the ridges, and the depth of the muscular depressions, indicate that the animal was an adult, which had long attained its full size. |
|  |  |  |  | Lydekker, 1876 | Found that there was not much difference in the dimensions of *G. sivalensis* vertebra and modern giraffes, and that some of the diameter measurements were even larger. *G. sivalensis* had similar molar teeth as extant giraffes, but with a neck about one third shorter. |
|  |  |  |  | Lydekker, 1883 | Lydekker corrected his previous postulates, saying that the extant giraffe used for comparison was immature and that comparisons were therefore invalid. Mentioned that the holotype was actually a 'fifth' cervical. |
|  |  | 39747 |  | Lydekker, 1885a | Described *G. sivalensis* as a very small individual. |
|  |  | B187 |  | Lydekker, 1885b | Mentioned a cast of the 'fifth' cervical vertebra located in the Indian Museum. |
|  | Second or third dorsal vertebra | 60 | Perim Island (referring most probably to Piram Bet Island, Gulf of Khambat | Falconer, 1868 | Second or third dorsal vertebra of giraffe, concave and convex articular surfaces present. Apophyses wanting. |
|  |  |  |  | Lydekker, 1883 | Referred to plate e of Fauna Antiqua Sivalensis. |
|  | Caudal fragment of "second" cervical vertebra |  | Perim Island (referring most probably to Piram Bet Island, Gulf of Khambat | (Falconer, 1845) | Adult. Well marked longitudinal ridge along middle, corresponding to the 'third' cervical. Same 'lateral ridges' which are different to extant giraffes. Ventral curve similar to C3 dimensions given. |
|  |  |  |  | Lydekker, 1883 | Lydekker called this specimen an imperfect fourth cervical vertebra. Also mentioned in the Palaeontological memoirs (Murchison, 1868b) p.391. May be the 'larger incomplete third cervical' from the Siwalik hills to which Lydekker referred (see below). |
|  |  | 39748 |  | Murchison, 1868a (plate E, Fig. 2) | Fragment of second cervical vertebra of *Giraffa (Camelopardalis)* *sivalensis*, from Perim Island. The right margin of the drawing shows the mesial longitudinal ridge under the side of the body, and the left margin is the ridge of the spinous process. The process pointing downwards on the left side is the inferior oblique process. The cup shaped articulating surface for the head of the third cervical vertebra is well seen. This specimen was in the collection of fossils brought from Perim Island by Captain Fulljames. Length of fragment, 4.9". Height of body posteriorly, 2.5". Greatest breadth posteriorly between remains of transverse processes, 3.1". Height of the spinal canal, 1.4". Height of the broken surface of the spine above inferior margin of body, 5.4". Vertical diameter of articulating cup, 2.1". Transverse diameter of articulating cup, 2.1" |
|  |  |  |  | Lydekker, 1883 | Referred to as the second specimen published in Falconer, 1845. |
|  |  |  |  | Lydekker, 1885a | Posterior part of a much battered fourth (?) cervical vertebra, intermediate in size between specimen 39746 and 39747. From the Siwaliks of ‘Perim Island in the Gulf of Cambay’. Cautley collection 1842. |
|  | Larger incomplete 'third' cervical |  | Siwalik Hills, India | Lydekker, 1883 | Mentioned that this specimen had not been described yet by Messrs Falconer and Cautley. |
|  | 'First' or ‘third’ cervical vertebra, imperfect |  | Siwalik hills, India | Murchison, 1868a (Unpublished plate E, Fig. 11) | Dimensions given. |
|  |  |  |  | Lydekker, 1883 | Seemingly as large as the corresponding bone of *G. camelopardalis*. Regarding the description of this specimen in the plates of the Fauna Antiqua Sivalensis (Murchison, 1868a), Lydekker mentioned that these measurements were probably erroneous. It is also noted that Falconer at this stage abandoned the distinction between *G. sivalensis* and *G. affinis.* |
|  |  | BM39746 |  | Lydekker, 1885a | Posterior moiety of the ‘third’ cervical vertebra of a full sized individual. Pliocene. Cautley collection 1842. |

| **Specimens ascribed to *G. sivalensis*** | **Fossil specimen:**  **Limb bones** | **Museum no** | **Origin** | **References to specimen** | **Paper summary or highlights** |
| --- | --- | --- | --- | --- | --- |
|  | Humerus, Right, two fragments | 43 | Perim Island (referring most probably to Piram Bet Island, Gulf of Khambat | Falconer, 1868 | From Perim Island. Lower articulating surface perfect, upper broken off immediately below the head. Exact form to that of the giraffe, but little larger. |
|  |  | 39749 | Siwalik hills, India | Lydekker, 1885a | Right humerus without proximal extremity of a small individual, believed to be from the Pliocene of the Siwaliks. |
|  |  | BM 37749 |  | Murchison, 1868a (unpublished plate E) |  |
|  |  |  |  | Lydekker, 1883 |  |
|  |  | B363 |  | Lydekker, 1885b | Cast of the greater portion of the right humerus. Original is from 'Siwalik hills' and is figured in The Fauna Antiqua Sivalensis (Murchison, 1868a) (unpublished plate E, Fig. 3). British museum no 39749. |
|  | Humerus, Left, proximal third | 17136 | Siwalik hills | Lydekker, 1885a | Proximal third of left humerus, from the Pliocene of the Siwalik hills. |
|  | Radius, Left, Upper portion | 690 | Siwalik | Falconer, 1868 | Bone much flattened, outer border considerable curve, abrupt expansion of the articulating head and sudden contraction of shaft. Nearly equal in dimensions to existing giraffe, but flattened. Greyish appearance due to sandstone matrix. |
|  |  |  |  | Lydekker, 1883 |  |
|  | Radius and Ulna, Left, Shaft only | 17130 | Siwalik | Lydekker, 1885a | From the Pliocene. Figured in Murchison (1868a) (Unpublished Plate E, Fig. 4) |
|  |  | BM 17130 |  | Murchison, 1868a (Unpublished plate E, Figs 4a and 4b) | Fragment of shaft of left radius and ulna  Length of fragment, 8.5 inch *(215.9 mm)*  Greatest diameter 3'' *(76.2 mm)*  Smaller diameter 2.1'' *(53.34 mm)*  Great diameter of ulna at upper extremity = 7'' *(177.8 mm)*  thickness of ulna at upper extremity = 5'' *(127 mm)* |
|  | Radius and ulna, restored |  |  | Falconer & Murchison (1867) (Unpublished plate E, Figs 5a and 5b) | No other info given |
|  | Metacarpus, L, lower end, with articulating surfaces | 52 | Perim Island (referring most probably to Piram Bet Island, Gulf of Khambat | Falconer, 1868 | Of the size of existing giraffe. |
|  | Metacarpus, fragment including upper end | BM39750 |  | Murchison, 1868a (plate E, Fig. 6) | Metacarpal bone, Fragment including upper end.  Length of fragment 18.7'' *(474.98 mm)*  Transverse diameter of upper extremity = 3.7'' *(93.98 mm)*  Antero-posterior diameter of upper extremity = 2.3'' *(58.42 mm)*  Transverse diameter of centre shaft = 2.4'' *(60.96 mm)*  Antero posterior diameter of centre shaft = 1.8’’ *(45.72 mm)* |
|  |  |  | Siwalik Hills | Lydekker, 1885a | Proximal half of left metacarpus, from the Pliocene. Presented 1842. |
|  | Metacarpus, fragment of shaft | BM17129 |  | Murchison, 1868a, (Unpublished plate E, Fig. 8) | Fragment of shaft of metacarpal bone  Length of fragment 6.8'' *(172.72mm).* |
|  |  |  |  | Lydekker, 1885a | From the Pliocene of the Siwaliks. |
|  | Metacarpus, fragment of shaft near lower end | BM17131 |  | Murchison, 1868a (Unpublished plate E, Fig. 9) | Fragment of shaft of metacarpal bone, near lower end  Length of fragment = 3.9'' *(99.06mm),*  Transverse diameter of shaft at lower extremity = 2.8'' *(71.12mm),*  Anterioposterior diameter of shaft at upper extremity = 1.5'' *(38.1mm)* |
|  |  |  |  | Lydekker, 1885a |  |
|  | Metacarpus, entire bone restored |  |  | Murchison, 1868a (Unpublished plate E, Figs 10a and 10b) | No other info provided. |
|  | Metacarpus, fragment of shaft | 39751 | Siwalik hills | Lydekker, 1885a | From the Pliocene. Cautley collection, presented in 1842. |
|  |  |  |  | Murchison, 1868a (Unpublished plate E Fig.7) |  |
|  | Phalangeals, proximal, two | 17131a | Siwalik hills | Lydekker, 1885a | These bones are almost indistinguishable from the corresponding bones of *G. camelopardalis*. Cautley collection 1842. |
|  | Phalangeal, proximal. | 17131b | Siwalik hills | Lydekker, 1885a | Closely resembling phalangeals marked 17131a, from the Pliocene. Cautley collection 1842. |
|  | Proximal anterior phalangeal | B362 | Punjab | Lydekker, 1885b |  |
|  | Proximal posterior phalangeal | B368 | Punjab | Lydekker, 1885b |  |
|  | Distal portion of tarsus metatarsus (imperfect inferiorly). And the first phalangeal | B186 | Punjab | Lydekker, 1885b |  |
|  | All *G. affinis* fossils |  |  | Lydekker, 1876 | Lydekker concluded that *G. sivalensis* and *G. affinis* are actually the same species, based on the characteristics of the fossil teeth on which *G. affinis* was founded. |
|  | Upper extremity of right metacarpus | 405 | Siwalik | Falconer, 1868 | Probably giraffe. Siwalik hills near Nahun (Nahan) |

| **Specimens ascribed to *G. sivalensis*** | **Fossil specimen:**  **Cranial specimens** | **Museum no** | **Origin** | **References to specimen** | **Paper summary or highlights** |
| --- | --- | --- | --- | --- | --- |
|  | Portion of L Maxilla with 3 molars |  |  | Lydekker, 1878, 1883 | The majority of these specimens do not differ in character from the corresponding teeth of the extant giraffe. Lydekker postulates that only *G. sivalensis* true upper molars are represent in this *Giraffa* molar catalogue. Nevertheless, he is open to the idea that there might have been a second or third species, based on the lower molars and premolars. All these specimens were assigned to *G. sivalensis* and 'some other species'. |
|  | Portion of R Maxilla containing the two last premolars (same individual as above?) |  |  |  | Lydekker (1883) expressed greater certainty that this and above specimens are part of the same animal. |
|  | Two detached penultimate molars |  |  |  |  |
|  | Two last right upper molars |  |  |  |  |
|  | Two penultimate upper molars |  |  |  |  |
|  | Last right upper premolar |  |  |  |  |
|  | Portion of R Maxilla containing the two last milk molars and first permanent molar |  |  |  |  |
|  | Fragment of left ramus of mandible containing the two last permanent molars |  |  |  |  |
|  | Another fragment of the right mandible containing the last tooth |  |  |  |  |
|  | First right mandibular molar |  |  |  |  |
|  | Last right mandibular premolar (large) |  |  |  |  |
|  | Penultimate left mandibular premolar |  |  |  |  |
|  | Fragment of right ramus of mandible containing two anterior premolars |  |  |  |  |
|  | Last left mandibular molar |  |  |  | Last molar only is too small for *G. sivalensis*, and probably belonged to another species. |
|  | Two last maxillary molars |  |  |  | These teeth had been collected, figured and described on plate 16 of Murchison, 1868b |
|  | Penultimate maxillary premolar |  |  |  |  |
|  | Three mandibular molars |  |  |  |  |
|  | Last mandibular premolar |  |  |  |  |
|  | Right ramus of the mandible with last premolar and the first and second true molars of a Siwalik giraffe |  |  | Lydekker, 1883 |  |
|  |  |  |  |  | In summary, up until Lydekker's (1883) publication: six mandibular specimens of Siwalik giraffes, of which five contained the last lower true molars and two contained both the second and first true molars and last premolar. Specimens are B179 (pl 16 Fig. 6), B179A; B178, Falconer's specimens, B173 (pl 16 Fig. 5), living giraffe, B1 (vol. 1 pl. 7 Fig. 14 and 16) |
|  | Lower jaw, right, three milk molars in site and first molar germ in jaw. Two posterior premolars. | 560 | Siwalik hills | Falconer, 1868 | There is netted rugosity of enamel – Therefore *Giraffa* and not Sivatherium or Bramatherium. (*Note however that Lydekker (1885a) proposed that a rugose enamel is a characteristic of the Giraffidae (which included the Sivatheres).* |
|  |  |  |  | Lydekker, 1883 | Lower jaws of a 'ruminant'. |
|  | Lower jaw, left, three milk molars | 561 | Siwalik hills | Falconer, 1868 | Enamel surface also shows rugose netting, but not clear enough to be confident. |
|  |  |  |  | Lydekker, 1883 | Two lower jaws of 'a ruminant'. |
|  | Part of lower jaw |  |  | Lydekker, 1876 |  |
|  | Molars | s560 & s561 |  | Falconer, 1868 |  |
|  |  |  |  | Lydekker, 1876 | These teeth are actually from a *Bos* *spp.* |
|  | Portions of the associated right and left maxillae, showing last 4 teeth | B184 | Asnot, Punjab | Lydekker, 1885b | Figured in Lydekker (1883) in plate xvi: "Collected by Mr Theobald in the Siwaliks of the Punjab". |
|  | Part of the maxilla with mm (milk molar?) 3, 4 and molar 1. | B177 | Potwar district, Punjab |  | Collected by Mr Theobald |
|  | Part of the right maxilla with m2, m 3 | B180 | Potwar district, Punjab |  | Collected by Mr Theobald. |
|  | Three detached maxillary true molars | B181 | Punjab |  | Collected by Mr Theobald. |
|  | Two detached maxillary premolars | B182 | Punjab |  | Collected by Mr Theobald. |
|  | Casts of the last maxillary and mandibular true molars | B183 | Siwalik hills |  | Originals are figured in Falconer & Cautley (1843) plate 2 Figs 4, 5 and in Murchison (1868b) pl. 16 Fig. 6 and 7. Originals in the British museum as 39756 and 39755 |
|  | Third left mandibular molar (?) | B346 | ? |  |  |
|  | Upper molar | B406 | Punjab |  |  |
|  | Part of right ramus of mandible | B173 | Niki, Punjab |  | Figured in Lydekker (1883) plate 16 Fig. 5. |
|  | Part of the right ramus of the mandible | B174 | Padri |  | Figured in Lydekker (1883) plate 7 Fig. 14. |
|  | Part of the left ramus of the mandible, with m2 and m3 | B178 | Potwar district, Punjab |  |  |
|  | Three specimens of the last mandibular true molars | B179 | Potwar district, Punjab |  | One of these is of very small size, and is figured in the Lydekker (1883) plate 16 Fig. 6. The author does not rule out the probability that this specimen really belonged to Palaeomeryx. |
|  | last left mandibular premolar | B185 | Asnot, Punjab |  |  |
|  | Cast of the last left mandibular premolar | B185a | Siwalik hills |  | Figured in Falconer & Cautley (1843) plate 2 Fig. 6 and in Murchison (1868b) Plate 16 Fig. 8, 9. |
|  | The last right mandibular milk molar | B175 | Asnot, Punjab |  | Figured in Lydekker (1883) plate 16 Fig. 8. |

| **Specimens ascribed to *G. affinis*, later placed under *G sivalenensis.*** | **Fossil specimens** | **Museum no** | **Origin** | **References to specimen** | **Paper summary or highlights** |
| --- | --- | --- | --- | --- | --- |
|  | Fragment of left maxilla including two rear molars. The ‘back part of the maxillary, beyond the teeth, is attached’. |  | Siwalik Hills. Described as a ‘soft fossil’ having been embedded in clay. | Falconer & Cautley, 1843 | Figured in Plate 2 fig. 3a and 3b of Falconer and Cautley (1843). Belonged to fully-grown individual. Specimen agrees almost exactly to that of an adult female giraffe. Dimensions:   - Joint length of two back molars, maxilla =2.5 inches. - Greatest with of last molar = 1.4 inches. - Greatest with of penultimate molar = 1.45 inches. |
|  |  | 39756 a | Siwalik Hills | Lydekker, 1885a | From the Pliocene. Left maxilla containing m2 and m3. Figured in Falconer & Cautley (1843) (above) and in Murchison (1868b) plate 16 Fig. 5. Cautley collection |
|  | Rear molar of right maxilla |  | Siwalik Hills. Described as a ‘soft fossil’ having been embedded in clay. | Falconer & Cautley, 1843 | Same form as left side (of 39756a?), but more worn - different individual? Figured in Plate 2 Fig 4 of Falconer & Cautley (1843). ‘The agreement extends down to the small cone of enamel at the base of the hollow between the barrels on the inside’ Dimensions:   - Length = 1.2 inch. - Width = 1.4 inch. |
|  |  | 39756 | Siwalik Hills | Lydekker, 1885a | ‘Third right upper true molar’. From the Pliocene of the Siwalik Hills. Figured in Falconer and Cautley, (1843) (above) and in Falconer and Murchison (1868b) vol 1 plate 16 Fig 6. Cautley collection. |
|  |  |  |  | Murchison, 1868b | Described as in Plate 16 fig 6 as ‘last upper molar, right side, one half of natural size’. |
|  | Fragment of left mandible containing the last molar |  | Siwalik Hills. Described as a ‘soft fossil’ having been embedded in clay. | Falconer & Cautley, 1843 | Same form and proportions of an extant female giraffe specimen observed by Falconer and Cautley (1843). Figured in plate 2 Fig 5a and 5b of Falconer and Cautley (1843). Dimensions of tooth:   - Length = 1.7 inch. - Greatest width = 1.0 inch. |
|  |  | 39755 | Siwalik Hills | Lydekker, 1885a | Described as a ‘third left lower true molar, in an early condition of wear’. From the Pliocene epoch. No mention is made of the ‘fragment of the left lower jaw’ noted by Falconer & Cautley (1843). |
|  |  |  |  | Murchison, 1868b | Volume 1 plate 16 figure 7. Described as last lower molar, left side. |
|  | Last premolar (‘false molar’) of the left mandible, detached. |  | Siwalik Hills. Described as a ‘soft fossil’ having been embedded in clay. | Falconer & Cautley, 1843 | Agrees closely with corresponding tooth in an extant female giraffe specimen observed by Falconer & Cautley. The specific tooth is noted to be ‘thicker’ in proportion to its length in giraffes compared to other ruminants. Figured in Plate 2 Fig 6 of Falconer & Cautley (1843). Dimensions:   - Length = 1.0 inches. - Width = 0.9 inches. |
|  |  | 39757 | Siwalik Hills | Lydekker, 1885a | Lydekker assigned this specimen as a ‘right lower premolar’, as opposed to Falconer and Cautley (1843) who considered it a ‘left’ premolar. From the Pliocene epoch. |
|  |  |  |  | Murchison, 1868b | Volume 1 plate 16 Fig 8. |
|  | Penultimate premolar of right maxilla |  | Siwalik Hills. Described as a ‘soft fossil’ having been embedded in clay. | Falconer & Cautley, 1843 | Described as a ‘false molar’. Except for ‘three tubercles at the inside of the base’ the tooth has the same size and form as the corresponding tooth in an extant female giraffe specimen observed by Falconer and Cautley. Figured in Plate 2 Fig 7 of Falconer and Cautley (1843), described here as: ‘second false molar; upper jaw, right side’. Dimensions given as:   - Length = 1.0 inch. - Breadth = 1.12 inch. |
|  |  |  |  | Murchison, 1868b | Plate 16, Fig 9. |
|  | First false molar of right maxilla |  |  | Falconer and Cautley, 1843 | Not figured in the referring text. Dimensions not given. |
|  |  |  |  | Lydekker, 1876 | Stated that based on erroneous teeth allocation by Falconer, there was probably not a *G. affinis* species. Appears to have been founded by mistake. |

References not included in main text document:

Lydekker R. 1885a. Catalogue of fossil mammalia. Part II. Containing the order ungulata, suborder Artiodactyla. London: Taylor and Francis. Printed by order of the Trustees.

Lydekker R. 1885b. Catalogue of the remains of Siwalik Vertebrata contained in the Geological Department of the Indian Museum, Calcutta. India: Printed by the Superintendent of Government Printing.
